# Assessment of Water Transport and Chemical Attack of Meta-Illite Calcined Clay Blended Cement in High-Performance Concrete

**DOI:** 10.3390/ma16227149

**Published:** 2023-11-13

**Authors:** David O. Nduka, Babatunde J. Olawuyi, Blas Cantero, Belén González-Fonteboa

**Affiliations:** 1 Department of Building Technology, College of Science and Technology, Covenant University, Km 10 Idiroko Road, Ota 12212, Ogun State, Nigeria; babatunde.olawuyi@covenantuniversity.edu.ng; 2 Department of Civil Engineering, School of Civil Engineering, Universidade da Coruña, 15179 A Coruña, Spain; belen.gonzalez.fonteboa@udc.es; 3 Department of Building, School of Environmental Technology, Federal University of Technology, Minna 92010, Niger State, Nigeria

**Keywords:** durability properties, high-performance concrete, meta-illite calcined clay, superabsorbent polymers

## Abstract

Rapid urbanisation causes a rise in the need for infrastructure, which in turn fuels the creation of additional concrete and further increases cement supplies. Activation of illite-based clay mineral and usage in concrete production is one of the sustainable ways to address the cement industry anthropogenic issues. This study evaluates the durability properties of water transport (water absorption, and capillary water absorption), and resistance to aggressive environments (5% solutions of hydrochloric acid, HCl; sodium sulphate, Na_2_SO_4_; and calcium chloride, CaCl_2_) of meta-illite calcined clay (MCC)-based high-performance concrete (HPC). For this purpose, concrete was produced with 5, 10, 15, 20, 25 and 30% MCC content in partial substitution of CEM II. Results from the water absorption tests indicate an average percentage value of 3.57%, 3.35% and 2.52% for all the observed mixes at 28, 56 and 90 days, respectively, with MCCC-10 HPC having an average best value of 2.23% across the curing ages. On all observed days, the 5 to 15% cement replacements had very close average water sorptivity value of 0.125 ± 0.001 mm/min^0.5^ with the control mix (0.113 ± 0.011 mm/min^0.5^). The aggressive environments exposure findings of the hardened MCC-based HPC specimens of 10 to 20% recorded an approximately 15% compressive strength loss in HCl, Na_2_SO_4_ and CaCl_2_ solutions over the 90 days of curing. In all, the HPC mixes of 5 to 15% MCC content obtained an average durability performance factor of 89%. As a result, these findings imply that MCC can replace cement in up to 15% of HPC production.

## 1. Introduction

Conventional concrete’s inability to withstand the damaging impact of environmental elements has received significant attention. Design Floor [1] following British and Indian standards grouped concrete grades into three distinct grades namely, normal (10–20 MPa), standard (25–45 MPa) and high strength (50–70 MPa) concrete. The application of normal and standard grades of concrete in heavy civil engineering projects and tall buildings can contribute to the fast deterioration of structural elements [2,3], reduce the service life and increase the life cycle cost of the entire concrete structure [2,4,5]. Ma et al. [6] and Hao et al. [7] pointed out that the incorporation of sustainable measures like the supplementary cementitious materials (SCMs) into concrete is one way of achieving resilience in infrastructure for next-generation projects. Therefore, developing quality concrete that will meet the requirements of weathering harmful climates, reducing deleterious materials’ ingress and preventing corrosion in reinforcement becomes critical. High-performance concrete (HPC) solves concrete durability issues due to its dense microstructure and packing density [8,9]. HPC is optimally achieved with a binary or a ternary mix of SCMs with Portland cement at a low water binder ratio (W/B) of <0.4 [10]. It is described as having a compressive strength and durability factor greater than 70–80 MPa and 80% respectively, a high cement content of 450–550 kg/m^3^ and a high dosage of superplasticiser of 5–15 lm/m^3^ [9]. Thus, the addition of SCMs in a blended cement matrix improves the durability dimensions of HPC.

Durability is one of the cardinal properties of hardened HPC that establishes its relevance in infrastructure and high-rise building projects. Zhou and Qiao [11] defined concrete and cementitious composite durability as maintaining the initial desired engineering properties without detecting degradation for ages in a harsh location. They likened concrete to an absorbent mixture susceptible to water and chemical ion penetrations. These permeating gases, liquids and chloride ions largely negatively influence concrete micro and macrostructures’ durability. The significant deterioration of concrete is classified into physical attacks (freeze-thaw and abrasion actions) and chemical attacks (sulphate attack and corrosion of reinforcement bars and steel fibres). Certain standard techniques for a detailed assessment of frost and chemical resistances of concrete are commonly used for durability evaluations. For example, ASTM C 1202 [12], ASTM C 666/C666M [13], and ASTM C 672/C672M [14], CEN/TR 15177 [15], SS 137244 [16] and CEN/TS 12390-9 [17] provide a standard test for the evaluation of chloride ion permeability resistance, freeze-thaw and scaling resistance, respectively. In addition, scanning electron microscopy (SEM) and X-ray computed tomography (CT) can detect micro-cracks and material deterioration.

The usefulness of calcined clay, especially metakaolin, in reducing the permeability of liquids and gases likely to allow the ingress of attacking ions in concrete has been a front-burner in concrete research. In this direction, Khan [18] pointed out the usefulness of SCM, like calcined clay in concrete production, to construct facilities like seafloor tunnels, offshore piers and platforms, highway bridges, sewage pipes and solid and liquid structures waste containing toxic chemicals and radiations in a hostile environment. Infrastructure projects of this magnitude require a much longer service life than expected from normal-strength concrete. Therefore, using SCMs like calcined clay in the concrete matrix refines the concrete products’ pore structure, improving durability and reducing the frequency and cost of replacement and repairs.

Researchers [19,20,21,22] have noted that other calcined clay minerals (illite, montmorillonite, smectite, etc.) are less investigated as SCMs due to the complexity of the clay mineral and lack of knowledge of the reaction mechanisms compared with kaolinite. Illitic clays have been documented to have a higher activation temperature than kaolin, and their pozzolanic reaction is slow compared with kaolinitic clays [20]. However, as posited by the authors, it is the most abundant type of clay in many parts of the world, making it a potential SCM in a cement-based matrix and reducing anthropogenic activities. The water demand and 28-day strength are unaffected by the addition of illitic calcined clay as a cement replacement, but the rate of strength development at an early age may be slowed down [18]. The early strength of concrete made with illite calcined clay is usually compromised due to the dilution effect at a higher replacement. However, the activation temperatures of 900 to 950 °C for illite clays have been proven to produce amorphous aluminosilicate after dehydroxylation and structural collapse [19,20,22].

Similarly, concrete and mortar durability properties made with illite, montmorillonite, smectite, etc., clays have recently been a subject for some researchers. For example, Vejmelková et al. [23] characterised calcined Czech claystone to determine tested HPC samples’ water absorption coefficient and diffusion resistance factor. The researchers replaced CEM I 52.5 R Portland cement with less than 2 µm-diameter size calcined Czech claystone thermally treated between 700 and 900 °C at 10–50% replacement levels. At 30% replacement, their results indicate an excellent resistance to liquid penetration and vapour transport in reducing much soluble salt-induced damage, freeze/thaw damage and possible infiltration of harmful gaseous substances. Also, Laidani et al. [24] evaluated the effect of calcined bentonite clay on the total porosity, chloride-ions penetration and gas permeability of self-compacting HPC. Their durability tests indicate an improved resistance to attacking ions with calcined bentonite as SCM. Again, Rossetti et al. [22] determined the efficacy of calcined illite clay blended cement in cement paste and mortar, respectively, exposed to sulphate attack. The authors examined the test specimen based on visual inspection, mass change, compressive strength, expansion and microstructural properties between 7 and 720 days. Their results showed a superior sulphate resistance of the mixtures with a high calcined illite clay content over limestone filler.

Finally, Msinjili et al. [25] assessed the chloride penetration resistance of low-grade kaolinitic clay in blended cement in mortars. Their results revealed that transport properties like chloride migration were reduced due to low-grade kaolinitic clay in the mortar mixtures. A reduced chloride penetration is associated with refining the pore structure of the tested samples with low-grade kaolinitic clay. The literature survey revealed that only a few investigations had been performed on HPC production. Specifically, there have been limited research efforts profiling the suitability of manufactured meta-illite calcined clay (MCC) in fulfilling the durability and strength long-term properties of HPC. Hence, this experimental investigation aims to assess the possibility of using MCC sourced from the southwestern Nigerian research institute as a binder replacement at an acceptable level of up to 30% for making HPC. The study incorporated a manufactured MCC of Nigerian origin in an HPC binary blend. The goal is to determine the durability properties like water absorption, capillary water absorption, and resistance to the aggressive chemical environments on HPC mixtures incorporating MCC as an SCM. The present paper draws on Nduka et al. [26], which studied the effect of MCC on HPC’s mechanical and microstructural properties. 

## 2. Materials and Methods

### 2.1. Materials

A CEM II B-L, 42.5 N Portland-limestone cement, manufactured by Dangote cement PLC, Lagos, Ogun State, Nigeria, and compliant with BS EN 197-1 [27] and DMS 29-1 [28] was used as the primary binder. A commercially available Nigeria Building and Road Research Institute (NBRRI) cement (calcined clay) serves as an SCM. Air-dried river sand with a minimum particle size of 300 µm and crushed granite stone as a coarse aggregate that passed a 13.50 mm BS standard sieve and retained at least a 9.50 mm sieve were used as aggregates under the requirement for aggregates specification for HPC production [29,30,31,32]. The physical and mechanical properties of the HPC constituents reproduced are displayed in Table 1. Masterglenium Sky 504, a polycarboxylic ether (PCE)-based superplasticiser (SP) supplied by BASF Limited, Lagos, Nigeria, was used in the manufacture of the concrete.

The chemical compositions of the CEM II and MCC were analysed employing X-ray fluorescence, XRF (Bruker AXS S4, explorer, Karlsruhe, Germany), and the results are presented in Table 2. 

Table 3 provides a breakdown of the mixed proportions of the HPC matrix. All HPC mixtures were quantified by weight using the British mix design method. The method presented five steps: (i) selection of compressive strength to determine the water-cement ratio. This step brought the concept of target strength to the forefront; (ii) determination of water content based on required workability while considering the influence of maximum aggregate size and type; (iii) determination of cement content; (iv) determination of aggregate content in total; and (v) determination of fine aggregate content from aggregate content in total. The first HPC mixture consists of CEM II only with other constituents’ materials. It is referenced as the control to achieve an HPC mix designed for a 28-day characteristic cube strength of 67 MPa incorporating an internal curing agent (SAP). The SAP absorption capacity determined by tea-bag test is shown in previous work [33]. The other mixtures comprise MCC tagged meta-illite calcined clay concrete (MCCC), which partially substitutes CEM II from 5% to 30% at 5% intervals at a permanent W/B of 0.3%, permanent SAP content of 0.3% b_wob_, and SP content (1.5% b_wob_). Extra water of 12.5 g/g of SAP was settled following the SAP absorbency established by the work of Olawuyi [34]. HPC ingredients were thoroughly prepared and mixed uniformly in a 120 litre pan-type concrete mixer. The workability of the HPC mixes and the HPC mortars’ setting times (initial and final) were measured following the BS EN 12350-5 [35] slump flow measurement technique and penetration resistance method under ASTM C 403 [36], respectively. The results of the two tests were reported in the earlier publication of Nduka et al. [26].

### 2.2. Specimen Preparation

Water absorption and chemical attack specimens were cast in 100 mm cubes, while the sorptivity of the HPC was evaluated using 100 Ø × 50 mm disc moulds. Specimens for sorptivity were cut from 100 Ø × 200 mm cylinders after demoulding using an electric handheld cutting machine. On an Impact, CN 155, vibrating table (Stevenston Ayrshire, UK), the casting was carried out in two layers and compacted for approximately 3 min. The samples were accurately distinguished for identification purposes, protected from moisture loss with a plastic sheet and demoulded 24 h after casting. The samples were cured again by full immersion in a room-temperature curing tank. The hardened samples for water absorption and sorptivity were tested after 28, 56 and 90 days of curing, respectively, and the triplicate average was recorded for each curing age. In contrast, the chemical-attack resistance hardened samples were evaluated after 7, 28, 56 and 90 days of curing.

### 2.3. Specimen Testing

#### 2.3.1. Water Absorption

The water absorption test followed EN, B.1097-6 [37]. The saturated HPC specimen’s weight, *M_wet_*, was first estimated and then dried in triplicate for 24 h at 110 ± 5 °C to a constant weight. The dry samples were measured and recorded as *M_dry_* after cooling. The weight of the saturated sample was subtracted from the weight of the dry sample, multiplied by 100 and the resulting weight was used to calculate the water absorption value. Thus, Equation (1) can mathematically express the water absorption (*WA*) value.

(1) $$WA=\frac{M_{wet}-M_{dry}}{M_{dry}}\times 100$$

#### 2.3.2. Sorptivity

**Figure 1:**
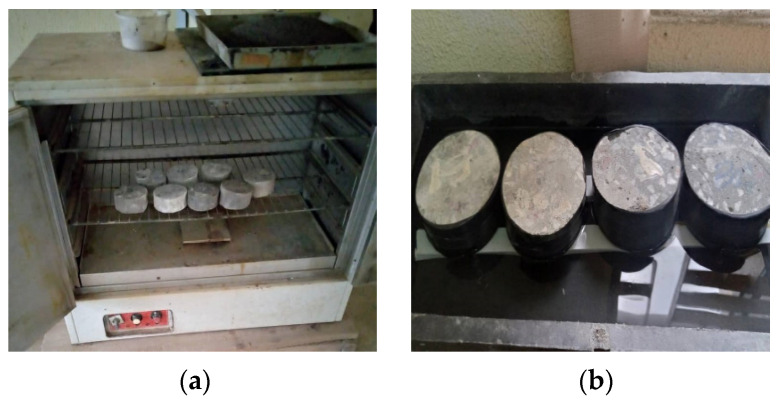
Sorptivity test setup: (**a**) drying of samples and (**b**) samples exposed to capillary action in the water.

Capillary water absorption measurements were conducted following the ASTM C 1585-04 [38] to evaluate the sorptivity coefficient of the HPC samples. Three-disc samples were first weighed for each HPC mixture and put into the oven at 110 ± 5 °C until a constant weight for 24 h. Then, the discs were weighed again, and the circumference was sealed with silicon tape to achieve a uniaxial water flow while the opposite faces were left open. Water was poured into a rectangular-shaped container up to the height of 30 mm, and samples were kept there for about 6 h. The data were recorded after 5, 10, 15, 30, 60, 120 and 180 min. The samples were then drawn and weighed once more to determine the quantity of water absorbed. Equation (2) below displays the results:

I = S × t^0.5^ (2)

where I is the total amount of water ingress measured in mm, S is the sorptivity factor, and t in min is the sample’s immersion time in the water. The volume was created by converting the mass of water absorbed by the specimen. A straight line resulted from plotting the water volume versus the square root of time. The slope of this straight line was used to calculate the sorptivity coefficient. After that, each HPC mix’s best-fit line was calculated using a regression analysis of the data points. The slope of the best-fit line represented the absorption rate of that mixture. The best-fitted line’s slope was the absorption rate for that mixture. The test setup for the sorptivity experiment is highlighted in Figure 1.

#### 2.3.3. Chemical Resistance

**Figure 2:**
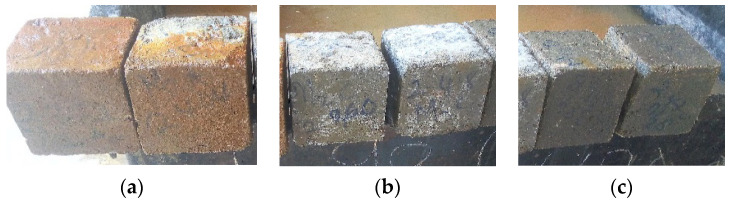
Samples of HPC hardened cubes exposed to chemical attacks: (**a**) HCl, (**b**) CaCl_2_ and (**c**) Na_2_SO_4_.

Solutions of 5% of hydrochloric acid (HCl), sodium sulphate (Na_2_SO_4_), and calcium chloride (CaCl_2_) were used for the resistance to aggressive chemical environment tests of the HPC samples, respectively. The 100 mm cube specimens of seven mixture formulations were stored for 90 days in 5% HCl, 5% Na_2_SO_4_ and 5% CaCl_2_ solutions. Throughout the testing period, open metallic containers were used while the pH of the acidic, chlorine, and sulphate environments was monitored. After determining the solutions’ pH every thirty days, the solutions were renewed. After 7 days, 28 days, 56 days and 90 days of exposure to aggressive chemical environments, the samples were removed and washed with water. The attached particles were then removed using a cloth napkin after the specimens were rapidly sun-dried. The effects of acid, chloride and sulphate attack were evaluated based on weight loss, followed by an evaluation of compressive strength. The compression test is conducted under BS EN 12390-3 [39] and RILEM Technical Recommendation TC14-CPC 4 [40]. Using a digital Materials Testing Machine (Model YES-2000, Eccles Technical Engineering Ltd., Salford, UK) with a maximum loading capacity of 2000 kN, 210 (100 mm) concrete cubes were examined. It was ensured that the face in contact with the loading platens differed from the cube’s cast face. Calculating the compressive strength (*f_c_*), Equation (3) was used.

(3) $$f_{c}=\frac{F}{A_{c}}$$

where *f*_c_ is the compressive strength of the HPC mixture in MPa; *F* is the maximum load at failure in kN; *A_c_* is the specimen area in mm. The 100 mm cube samples exposed to aggressive environments are shown in Figure 2.

## 3. Results and Discussion

### 3.1. Water Absorption

**Figure 3:**
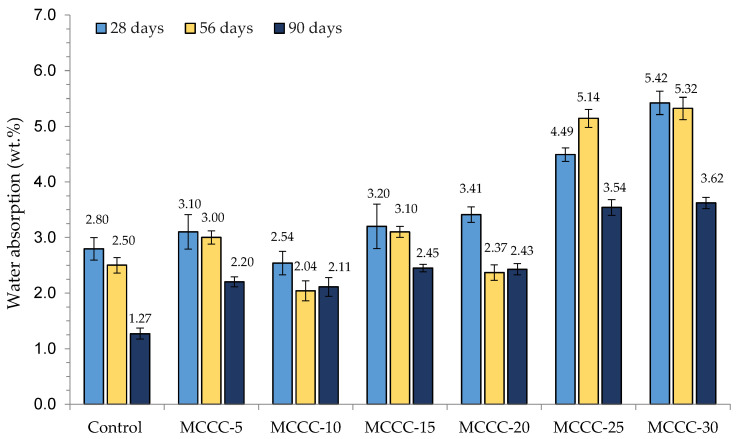
Water absorption development of HPCs incorporating MCC at different curing days.

The water absorption development of the HPCs produced with MCC cured for 28, 56 and 90 days is shown in Figure 3. As seen from the figure, the values of this test ranged between 2.54 and 5.42% for 28 days, 2.04 and 5.32% for 56 days, and 1.27 and 3.62% for 90 days. At 28 days of curing, the MCCC-10 mix had the lowest water absorption value of 2.54%, followed by the control (2.80%) with 0% MCC content.

Other mixtures at this age had higher water absorption values corresponding to 3.10, 3.20, 3.38, 3.41, 4.49 and 5.42% for MCCC-5, MCCC-15, MCCC-20, MCCC-25 and MCCC-30, respectively, than control. At 56 days, the MCCC-10 and MCCC-20 water absorption decreased significantly over the control (2.50%), with MCCC-10 having the best value of 2.04% at this age. The water absorption at 90 days showed a similar trend with 28 and 56 days. The control exhibited the best water absorption of 1.27% at this age, followed by MCCC-10 (2.11%) with a marginal difference from the control. The variation in results found here may have been impacted by the degree of pores, consequently influencing absorption tendencies. Adding MCC in the HPC mixtures moderately improves the water absorption capacities of some tested mixtures. The results agree with Laidani et al. [24] on less water absorption in high-strength concrete incorporating clay as a pozzolan. MCC-blended cement functions as a filler by producing additional C-S-H gel with Ca(OH)_2_ aiding in refining the porosity of the binders’ capillary. Laidani et al. [24] also found that the blend of calcined illitic mineral-based clay in self-compacting concrete reduces the porosity accessible to water. Comparing these values with ASTM C 642 [41] is encouraging, placing the acceptable water absorption range for HPC within 2–5%.

### 3.2. Sorptivity

Table 4 shows the effects of time on variations in water absorption by capillary pressure. For MCC-blended HPCs, the absorption rate (sorptivity coefficient) table was created by dividing the change in weight per unit area by the unit weight of water and the square root of the time (minutes) as recommended in ASTM C 1585-04 [41].

The table depicts blended MCC cement’s influence on HPC’s water sorptivity capacity at 28, 56 and 90 days. As a result of microstructural behaviour coupled with uninterrupted cement hydration, the water ingress varies across the curing ages for each mix. At 28 days, the MCCC-30 mixture exhibited the greatest water sorptivity of 0.190 ± 0.020 mm/min^0.5^ over the 180 min inspection period. At the same age, the control HPC had the lowest water capillary action of 0.128 ± 0.010 mm/min^0.5^. Among the MCC- induced HPC at 28 days, MCCC-10 had the lowest water sorptivity of 0.130 ± 0.014 mm/min^0.5^. At 56 days, a decreased water ingress trend could be observed in all the mixes. Compared with the control mix (0.113 ± 0.014 mm/min^0.5^), the MCCC-5 to MCCC-10 blended HPCs had average water sorptivities of 0.125 ± 0.015 mm/min^0.5^, highlighting a 0.012 ± 0.001 mm/min^0.5^ difference. Similar trends continued for 90 days; the average water ingress sorptivities for MCCC-5 to MCCC-15 blended HPCs was 0.106 ± 0.013 mm/min^0.5^, slightly higher than the control mix. The MCCC-5 had the lowest water absorption value of 0.101 ± 0.011 mm/min^0.5^ at this age. On all observed days, the 5%, 10% and 15% cement replacements had close water sorptivity values with the control mix. These results may be attributed to the physical properties regarding the greater specific surface area and pore size distribution of MCC. It is anticipated that adding MCC at an optimised content to concrete will densify the transition zone between aggregates and the cement matrix and refine the pore structures of the bulk paste. The findings of this study correspond well with those of Vejmelková et al. [23], who investigated the water absorption coefficient of calcined clay-based HPC to determine the durability of their tested samples.

The correlation coefficient of MCC-modified HPC at 28, 56 and 90 days is also shown in Table 4. At 28 days, the correlation coefficient between the rate of absorption and the square root of time for all mixtures was 0.904–0.984, while at 56 and 90 days, the correlation coefficients were 0.927–0.991 and 0.927–0.991, respectively. This result indicates that MCCC-10 showed a higher reliability (R^2^= 0.984, 28 d; R^2^= 0.952, 56 d and R^2^= 0.988, 90 d) across the observation days than other tested mixtures as specified in the standard (R^2^= 0.98).

**Figure 4:**
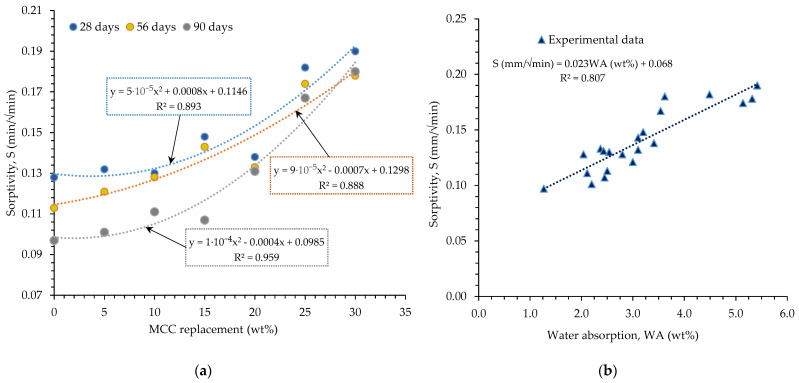
(**a**) Sorptivity and MCC content relationship, and (**b**) sorptivity and water absorption relationship.

Figure 4 show the relationship between HPC sorptivity and MCC content and water absorption, respectively. The relationship between HPCs’ sorptivity and MCC content is depicted in Figure 4a. As the figure shows, the relationship between the two properties was observed to be linear up to 15% MCC content and quadratic from 20% MCC content and above. There is a high correlation coefficient for 90 days of hydration (R^2^ > 0.96), while 56 and 28 days of observation attested to moderate correlation coefficients of 0.89, respectively. A greater porosity, pore system connection and dilution effect increased higher MCC-content HPCs’ sorptivity, which directly contributed to the stringent non-linearity observed in the MCCC-20 to MCCC-30 mixtures. In the graph in Figure 4b, it was discovered that sorptivity was directly correlated with the water absorption coefficient (R^2^ = 0.81). 

### 3.3. Chemical Durability: Acid, Sulphate and Chlorine Attacks on HPC

#### 3.3.1. Weight Changes

The influence of MCC on the chemical durability of HPCs assessed by weight drops due to chemical attacks was systematically evaluated. Table 5 shows the weight changes (WC) reflections for MCC-based HPC after 7, 28, 56 and 90 days of exposure to plain water (H_2_O), acid (HCl), sulphate (Na_2_SO_4_) and chlorine (CaCl_2_) environments. The control mixture showed a weight change of −0.5, −4.7, −5.5 and −7.4% for HCl at 7, 28, 56 and 90 days, respectively, compared to water curing. The weight changes in HPCs exposed to chemical environments varied from −0.5 to −10.4%, −2.4 to −6.1% and −0.9 to −6.4% for acid, sulphate and chlorine environments, respectively.

Comparing different chemical curing environments with this mixture shows that all the HPC samples demonstrated insignificant weight changes across the curing ages. The table shows that the various MCC-blended mixtures (MCCC-5 to MCCC-30) demonstrated minimal weight reductions of less than 10% in the different chemical environment exposures across the curing ages. The maximum weight reduction of 10% was scantily recorded in higher CEM II replacement with MCC exposed to an acidic environment at 90 days of observation. The various chemical environment exposures merely tampered with the binder phase, calcium hydroxide and C-S-H gel. These findings show that, unlike conventional concrete, HPCs modified with meta-illite clay are less susceptible to chemical attacks following their dense microstructure and packing density [4]. Therefore, the results agree with Kwan and Wong’s [42] idea of HPC’s ability to withstand chemical attacks over several years.

#### 3.3.2. Compressive Strength Analysis

**Figure 5:**
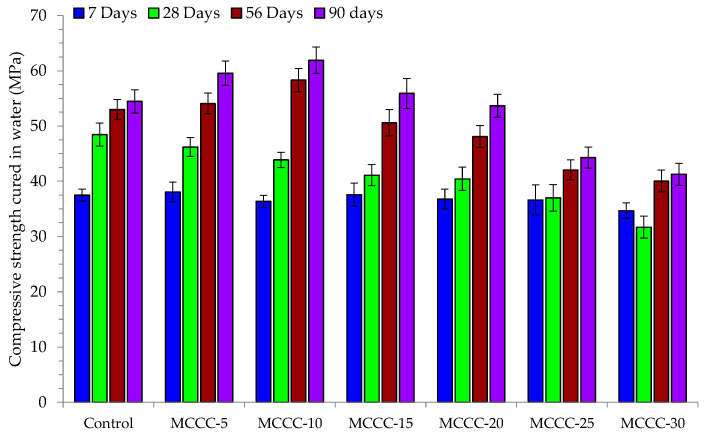
Compressive strength of MCC-blended HPC cured in water.

**Figure 6:**
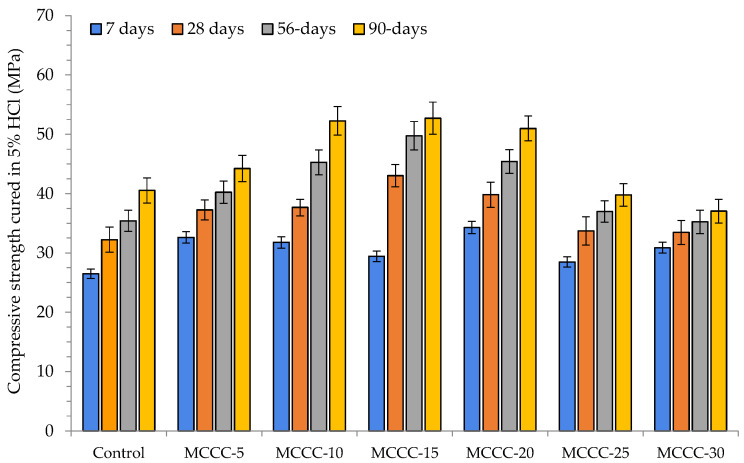
Compressive strength of MCC-blended HPC cured in 5% HCl.

**Figure 7:**
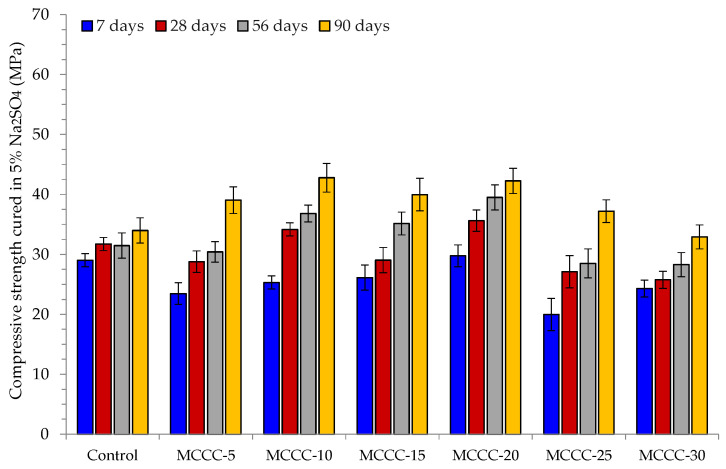
Compressive strength of MCC-blended HPC cured in 5% Na_2_SO_4_.

**Figure 8:**
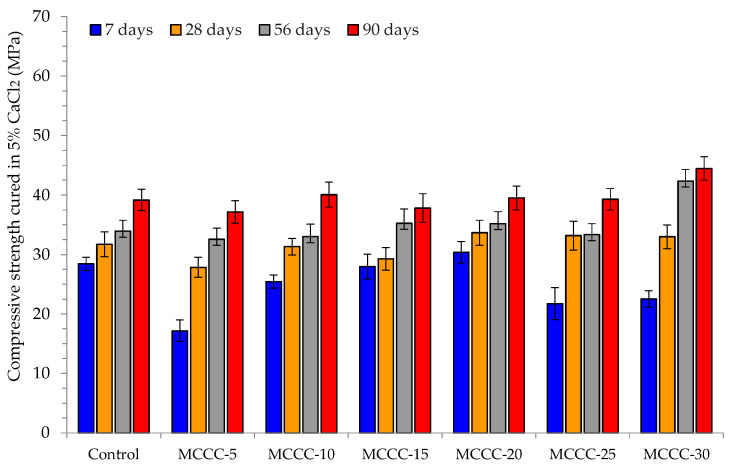
Compressive strength of MCC-blended HPC cured in 5% CaCl_2_.

Figure 5 presents the compressive strength of control and the various MCC-blended HPC samples cured in water for 7, 28, 56 and 90 days. On the other hand, Figure 6, Figure 7 and Figure 8 showed the influence of HCl, Na_2_SO_4_ and CaCl_2_ attacks after 7, 28, 56 and 90 days for the control, MCCC-5, MCCC-10, MCCC-15, MCCC-20, MCCC-25 and MCCC-30 mixtures, respectively. The compressive strength values of the control and MCC-blended HPC mixtures cured in the aggressive environments were compared with the plain water curing for all the observation days. The control sample recorded 26.45, 32.92, 35.42 and 40.54 MPa; 29.02, 31.72, 31.46 and 33.99 MPa and 28.44, 31.71, 33.93 and 39.15 MPa in HCl, Na_2_SO_4_ and CaCl_2_ solutions at 7, 28, 56 and 90 days testing periods. As the curing ages increase, the HPC control’s compressive strength increases.

The chlorine environment relatively strongly impacted the tested samples in this mixture. The figure shows that the MCC-blended HPC samples showed the highest compressive strength reductions between 15.20 and 27.41%, mostly in MCCC-5, MCCC-25 and MCCC-30 replacements across all tested ages in the chemical environments. The staggering loss of compressive strength values obtained in the various replacements may be due to vibration inconsistencies of the tested cubes and high cement content replacements with MCC. The remaining mixes (MCCC-10, MCCC-15 and MCCC-20) recorded reduced compression strength variation from a minimum of 1 to a maximum of 14.59%, indicating that ~15% strength was lost in HCl, Na_2_SO_4_ and CaCl_2_ over the 90 days of curing. Most of the HPC sample’s strength was lost in the acidic environment compared to sulphate and chlorine environments. Nikookar et al. [43] found that a cementitious matrix interaction with a low chlorine content produces Friedel’s salt and bonded calcite within the mix, which probably improves the microstructure by filling up voids. In the same vein, Hao et al. [7] observed that SCMs in concrete reduce the development of ettringite and gypsum which causes concrete expansion. This development stems from the ability of SCMs like MCC to reduce calcium hydroxide coming from hydration leading to less calcium hydroxide reaction with sulphate ions. The findings conformed to Vivek and Dhinakaran’s [44] report on the breakdown of cement paste and aggregate links due to C-S-H interference with acid. All the MCC-tested samples exhibited a compressive strength increase irrespective of the chemical curing environment at all curing ages.

## 4. Conclusions

This research examined the underlying durability properties of MCC as an SCM in a binary blended cement for HPC production. The study’s findings are restricted to MCC mineral and CEM II binders utilised in the HPC. Due to the distinct microstructural reactions of MCC with cement, the results may differ if alternative categories of kaolinite and montmorillonite-based mineral clays are utilised. The data presented here allow for the following conclusion to be made:

- According to ASTM C 642 [41], the water absorption values obtained in MCC-based HPCs are within the 2 to 5% acceptable range of water absorption for HPC.
- On all observed days, the 5–15% cement replacements had very close water sorptivity values attributable to the physical properties regarding the greater specific surface area and pore size distribution of MCC.
- The relationship between HPCs sorptivity and MCC content highlighted a high correlation coefficient for 90 days of hydration (R^2^ > 0.96), while 56 and 28 days observation attested to moderate correlation coefficients of 0.89, respectively. In the same vein, MCC replacement and the water absorption relationship depicted a positive relationship with the water absorption (R^2^ = 0.81).
- At 56 and 90 days curing ages, the HPC specimen MCCC-10 had the highest compressive strength. The 10% replacement mix (MCCC-10) had the highest compressive strength across all curing ages, indicating that MCC is best for realising HPC at this level of CEM II replacement.
- The various MCC-blended mixtures (MCCC-5 to MCCC-30) demonstrated minimal weight reductions of less than or equal to 10% in the different chemical environment exposures across the curing ages.
- The HPC mixes (MCCC-10, MCCC-15 and MCCC-20) recorded reduced compression strength variation in the 1% to 14.59% range, indicating that ~15% strength was lost in HCl, Na_2_SO_4_ and CaCl_2_ over the 90 days of curing.
- For all mix types, the compressive strength of the tested samples was most affected by the acidic environment with approximately 15% strength lost in HCl, Na_2_SO_4_ and CaCl_2_ over the 90 days of curing.

## Figures and Tables

**Table 1:** Physical and mechanical properties of binders and aggregates.

Material Properties	MCC	CEM II	Sand	Coarse Aggregate
Fineness modulus	-	-	2.87	-
Specific gravity	2.81	3.12	2.65	2.7
Water absorption, %	-	-	1.44	1.26
Aggregate crushing value, %	-	-	-	28
Aggregate impact value, %			-	11
BET SSA (m^2^/g) MultiPoint	4.649 × 10^2^	8.182 × 10^2^	-	-
Pore diameter mode—DA (nm)	2.88	2.92	-	-
Particle size distribution of binders				
D_90_ (µm)	42.35	48.00		
D_50_ (µm)	15.40	20.05		
D_10_ (µm)	2.04	1.48		

**Table 2:** Oxide composition of binder constituents [26].

Oxides	SiO_2_	Al_2_O_3_	Fe_2_O_3_	CaO	MgO	K_2_O	P_2_O_5_	MnO	LOI
**MCC (%)**	60.92	3.20	3.43	1.83	0.24	0.35	0.18	0.12	7.68
**CEM II (%)**	15.38	4.14	3.19	56.92	2.44	0.21	0.28	0.04	15.59

**Table 3:** Mix constituents of HPC with MCC [26].

Constituents	Mixes (kg/m^3^)						
	Control	MCCC-5	MCCC-10	MCCC-15	MCCC-20	MCCC-25	MCCC-30
Water	156	156	156	156	156	156	156
Cement	540	513	486	459	432	405	378
MCC	0	27	54	81	108	135	162
Coarse aggregate	1050	1050	1050	1050	1050	1050	1050
Sand	700	700	700	700	700	700	700
SAP (0.3% b_wob)_	1.62	1.62	1.62	1.62	1.62	1.62	1.62
SP (1.5% b_wob_)	8.10	8.10	8.10	8.10	8.10	8.10	8.10
Water/binder (W/B) *	0.3	0.3	0.3	0.3	0.3	0.3	0.3
Additional water	20.30	20.30	20.30	20.30	20.30	20.30	20.30

* W/B = ((water + liquid content of SP)/(cement + MCC).

**Table 4:** Sorptivity characteristics of MCC-based HPCs at 28, 56 and 90 days (±, standard deviation).

HPC Mix	28 Days		56 Days		90 Days	
	S (mm/min^0.5^)	R^2^	S (mm/min^0.5^)	R^2^	S (mm/min^0.5^)	R^2^
Control	0.128 ± 0.010	0.941	0.113 ± 0.014	0.916	0.097 ± 0.011	0.982
MCCC-5	0.132 ± 0.011	0.965	0.121 ± 0.012	0.947	0.101 ± 0.011	0.926
MCCC-10	0.130 ± 0.014	0.984	0.128 ± 0.018	0.952	0.111 ± 0.012	0.988
MCCC-15	0.148 ± 0.016	0.921	0.143 ± 0.013	0.941	0.107 ± 0.015	0.927
MCCC-20	0.138 ± 0.015	0.945	0.133 ± 0.010	0.921	0.131 ± 0.014	0.955
MCCC-25	0.182 ± 0.014	0.904	0.174 ± 0.014	0.988	0.197 ± 0.021	0.971
MCCC-30	0.190 ± 0.019	0.912	0.178 ± 0.020	0.941	0.180 ± 0.020	0.991

**Table 5:** Effect of MCC-blended cement on HPC weight change in plain water, acid, sulphate and chlorine environments.

HPC Mix	Control	MCCC-5	MCCC-10	MCCC-15	MCCC-20	MCCC-25	MCCC-30
H_2_O curing (WC%)							
7 days	7.3	7.5	7.4	7.8	7.8	8.1	8.7
28 days	9.2	9.4	9.7	9.6	10.4	10.8	11.1
56 days	12.7	13.1	13	13.4	13.7	13.9	14.1
90 days	13.7	13.9	13.7	14.5	14.9	15.2	15.4
**5% HCL curing (WC%)**							
7 days	−0.5	−0.9	−1.1	−1.4	−1.7	−2.2	−2.4
28 days	−4.7	−5.1	−5.4	−5.9	−5.9	−6.4	−6.7
56 days	−5.5	−6.2	−6.7	−6.6	−6.9	−7.4	−8.1
90 days	−7.4	−7.8	−8.4	−8.7	−8.9	−9.9	−10.4
**5% Na_2_SO_4_ curing (WC%)**							
7 days	1.4	1.7	1.7	2.1	2.3	2.4	2.6
28 days	−2.4	−2.8	−2.8	−2.9	−3.1	−3.7	−3.9
56 days	−3.8	−3.9	−4.1	−4.0	−4.4	−4.8	−4.9
90 days	−4.9	−5.4	−5	−5.1	−5.7	−5.9	−6.1
**5% CaCl_2_ curing (WC%)**							
7 days	0.9	1.1	1.4	1.9	2.1	2.1	2.5
28 days	−2.7	−2.9	−3.1	−3.4	−3.9	−4.4	−5.1
56 days	−3.9	−4.1	−4.4	−3.9	−4.2	−5.1	−5.9
90 days	−5.1	−5.2	−5.3	−5.4	−5.4	−5.9	−6.4

## Data Availability

Data will be made available on request.

## References

[1] (2023). Design Floor. Different Concrete Grades Based on Different International Codes. https://designfloor.org/concrete-grades/.

[2] Zhang Y., Zhu P., Liao Z., Wang L. (2020). Interfacial bond properties between normal strength concrete substrate and ultra-high performance concrete as a repair material. Constr. Build. Mater..

[3] Oyebisi S., Ede A., Olutoge F., Omole D. (2020). Geopolymer concrete incorporating agro-industrial wastes: Effects on mechanical properties, microstructural behaviour and mineralogical phases. Constr. Build. Mater..

[4] Li J., Wu Z., Shi C., Yuan Q., Zhang Z. (2020). Durability of ultra-high performance concrete—A review. Constr. Build. Mater..

[5] Nduka D.O., Olawuyi B.J., Joshua O.O., Omuh I.O. (2022). A Study on Gel/Space Ratio Development in Binary Mixture Containing Portland Cement and Meta-Illite Calcined Clay/Rice Husk Ash. Gels.

[6] Ma D., Zhang M., Cui J. (2023). A review on the deterioration of mechanical and durability performance of marine-concrete under the scouring action. J. Build. Eng..

[7] Hao H., Bi K., Chen W., Pham T.M., Li J. (2023). Towards next generation design of sustainable, durable, multi-hazard resistant, resilient, and smart civil engineering structures. Eng. Struct..

[8] Sohail M.G., Kahraman R., Al Nuaimi N., Gencturk B., Alnahhal W. (2020). Durability characteristics of high and ultra-high performance concretes. J. Build. Eng..

[9] Dushimimana A., Niyonsenga A.A., Nzamurambaho F. (2021). A review on strength development of high performance concrete. Constr. Build. Mater..

[10] Nduka D.O., Olawuyi B.J., Fagbenle O.I., Fonteboa B.G. (2022). Assessment of the Durability Dynamics of High-Performance Concrete Blended with a Fibrous Rice Husk Ash. Crystals.

[11] Zhou Z., Qiao P. (2018). Durability of ultra-high-performance concrete in tension under cold weather conditions. Cem. Concr. Compos..

[12] (2019). Standard Test Method for Electrical Indication of Concrete’s Ability to Resist Chloride Ion Penetration.

[13] (2015). Standard Test Method for Resistance of Concrete to Rapid Freezing and Thawing.

[14] (2003). Standard Test Method for Scaling Resistance of Concrete Surfaces Exposed to Deicing Chemicals.

[15] (2006). Technical Report of Testing the Freeze Thaw Resistance of Concrete Internal Structural Damage.

[16] (2005). Standard Concrete Testing Hardened Concrete Scaling at Freezing.

[17] (2006). Technical Specifications of Testing Hardened Concrete-Part 9: Freeze-Thaw Resistance–Scaling.

[18] Khan M.I. (2012). Mix proportions for HPC incorporating multi-cementitious composites using artificial neural networks. Constr. Build. Mater..

[19] Garg N., Skibsted J. (2016). Pozzolanic reactivity of a calcined interstratified illite/smectite (70/30) clay. Cem. Concr. Res..

[20] Irassar E.F., Bonavetti V.L., Castellano C.C., Trezza M.A., Rahhal V.F., Cordoba G., Lemma R. (2019). Calcined illite-chlorite shale as supplementary cementing material: Thermal treatment, grinding, color and pozzolanic activity. Appl. Clay Sci..

[21] Marchetti G., Rahhal V., Pavlík Z., Pavlíková M., Irassar E.F. (2020). Assessment of packing, flowability, hydration kinetics, and strength of blended cements with illitic calcined shale. Constr. Build. Mater..

[22] Rossetti A., Ikumi T., Segura I., Irassar E.F. (2021). Sulfate performance of blended cements (limestone and illite calcined clay) exposed to aggressive environment after casting. Cem. Concr. Res..

[23] Vejmelková E., Keppert M., Rovnaníková P., Ondráček M., Keršner Z., Černý R. (2012). Properties of high performance concrete containing fine-ground ceramics as supplementary cementitious material. Cem. Concr. Compos..

[24] Laidani Z.E.A., Benabed B., Abousnina R., Gueddouda M.K., Kadri E.H. (2020). Experimental investigation on effects of calcined bentonite on fresh, strength and durability properties of sustainable self-compacting concrete. Constr. Build. Mater..

[25] Msinjili N.S., Vogler N., Sturm P., Neubert M., Schröder H.-J., Kühne H.-C., Hünger K.-J., Gluth G.J. (2021). Calcined brick clays and mixed clays as supplementary cementitious materials: Effects on the performance of blended cement mortars. Constr. Build. Mater..

[26] Nduka D.O., Olawuyi B.J., Fagbenle O.I., Fonteboa B.G. (2021). Effect of K_y_Al_4_(Si_8_-y)O_20_(OH)_4_ Calcined Based-Clay on the Microstructure and Mechanical Performances of High-Performance Concrete. Crystals.

[27] (2011). Cement–Part 1: Composition, Specifications and Conformity Criteria for Common Cements.

[28] (2018). Composition, Specification and Conformity Criteria for Common Cements.

[29] Neville A.M. (2012). Properties of Concrete.

[30] Zeyad A.M., Johari M.A.M., Abutaleb A., Tayeh B.A. (2021). The effect of steam curing regimes on the chloride resistance and pore size of high–strength green concrete. Constr. Build. Mater..

[31] Zeyad A.M., Johari M.A.M., Alharbi Y.R., Abadel A.A., Amran Y.M., Tayeh B.A., Abutaleb A. (2021). Influence of steam curing regimes on the properties of ultrafine POFA-based high-strength green concrete. J. Build. Eng..

[32] Arulmoly B., Konthesingha C., Nanayakkara A. (2021). Performance evaluation of cement mortar produced with manufactured sand and offshore sand as alternatives for river sand. Constr. Build. Mater..

[33] Olawuyi B.J., Boshoff W. (2017). Influence of SAP content and curing age on air void distribution of high performance concrete using 3D volume analysis. Constr. Build. Mater.

[34] Olawuyi B.J. (2016). The Mechanical Behaviour of High-Performance Concrete with Superabsorbent Polymers (SAP). Ph.D. Thesis.

[35] (2009). Testing Fresh Concrete Flow Table Test.

[36] (2008). Standard Test Method for Time of Setting of Concrete Mixtures by Penetration Resistance.

[37] (2013). Tests for Mechanical and Physical Properties of Aggregates Part 6: Determination of Particle Density and Water Absorption.

[38] (2004). Standard Test Method for Measurement of Rate of Absorption of Water by Hydraulic Cement Concretes.

[39] (2019). Testing Hardened Concrete. Compressive Strength of Test Specimens.

[40] International Union of Testing and Research Laboratories for Materials and Structures (RILEM) (1994). CPC4—Compressive Strength of Concrete (1975). TC14-CPC, RILEM Technical Recommendations for the Testing and Use of Construction Materials.

[41] (2006). Standard Test Method for Density, Absorption, and Voids in Hardened Concrete.

[42] Kwan W.H., Wong Y.S. (2020). Acid Leached Rice Husk Ash (ARHA) in Concrete: A Review. Mater. Sci. Energy Technol..

[43] Nikookar M., Brake N.A., Adesina M., Rahman A., Selvaratnam T., Snyder H.A., Günaydın-Sen O. (2022). Reutilization of oil and gas produced water in cement composite manufacturing. J. Clean. Prod..

[44] Vivek S.S., Dhinakaran G. (2017). Durability Characteristics of Binary Blend High Strength SCC. Constr. Build. Mater..

